# *Operando* spatiotemporal super-resolution of thermal events monitoring in lithium metal batteries

**DOI:** 10.1093/nsr/nwaf088

**Published:** 2025-03-06

**Authors:** Chonghao Zhang, Zecong Liu, Zhoujie Lao, Yuting Zhou, Xiao Xiao, Tao Feng, Chengshuai Chang, Ruohui Wang, Guangmin Zhou, Xun Guan

**Affiliations:** Tsinghua Shenzhen International Graduate School, Tsinghua University, Shenzhen 518055, China; Tsinghua Shenzhen International Graduate School, Tsinghua University, Shenzhen 518055, China; Tsinghua Shenzhen International Graduate School, Tsinghua University, Shenzhen 518055, China; Tsinghua Shenzhen International Graduate School, Tsinghua University, Shenzhen 518055, China; Tsinghua Shenzhen International Graduate School, Tsinghua University, Shenzhen 518055, China; Tsinghua Shenzhen International Graduate School, Tsinghua University, Shenzhen 518055, China; Tsinghua Shenzhen International Graduate School, Tsinghua University, Shenzhen 518055, China; Guangdong Provincial Key Laboratory of New Energy Materials Service Safety, College of Materials Science and Engineering, Shenzhen University, Shenzhen 518060, China; School of Physics, Northwest University, Xi'an 710127, China; Tsinghua Shenzhen International Graduate School, Tsinghua University, Shenzhen 518055, China; Tsinghua Shenzhen International Graduate School, Tsinghua University, Shenzhen 518055, China

**Keywords:** lithium metal batteries, spatiotemporal, super-resolution, thermal monitoring

## Abstract

Safety challenges in high-capacity lithium metal batteries primarily arise from thermal runaway, leading to smoke emissions, fires or explosions. Real-time monitoring of internal temperature distribution is necessary to ensure safe operation and enhance cell performance. However, current methods lack dimensionality, precision, and timeliness, hindering the detection of uneven lithium deposition and localized temperature variations that drive capacity fade and safety risks. Here, we develop an *operando* spatiotemporal super-resolution thermal monitoring system capable of real-time, super-resolution temperature mapping across the lithium anode with a record-high spatial resolution of 1820 points cm^−^², and a temporal resolution of 1 frame per 3 seconds. Utilizing optical frequency-domain reflectometry, an Archimedean spiral fiber configuration, and super-resolution algorithms, we capture critical thermal variations and identify hotspots during cycling. To improve thermal uniformity and reduce safety risks, we apply protective strategies, including pyramid patterning, copper mesh and polylactic acid. Cells with these measures, as monitored by our system, show reduced average temperatures, delayed capacity degradation and fewer hotspots. This innovative monitoring approach not only integrates cutting-edge optical technology with energy storage diagnostics but also establishes a robust framework for assessing thermal management strategies, thus significantly advancing the safety and energy density of lithium metal batteries for sustainable energy applications.

## Teaser

This study presents the first operando spatiotemporal super-resolution thermal monitoring system for lithium metal batteries, offering unprecedented spatial and temporal precision to enhance safety, verify capacity degradation and optimize thermal management strategies.

## INTRODUCTION

As the global transition to clean energy accelerates, there is an increasing demand for innovative energy storage technologies. Lithium metal electrodes are emerging as a transformative solution, offering a theoretical capacity an order of magnitude higher than conventional lithium-ion batteries [1,2]. This significant advancement positions lithium metal batteries (LMBs) as a cornerstone for achieving the high energy densities that will be required by future technologies [3]. However, the practical deployment of these batteries is hindered by critical safety concerns [4]. The degradation and safety show strong temperature dependence [5], particularly the risk of thermal runaway [6], which results in severe fires and explosions. Rigorous thermal monitoring throughout the entire life cycle of LMBs is essential to address these challenges. By mitigating safety risks while capitalizing on the high capacity of lithium metal, such efforts will enable the development of the next generation of high-energy-density, resource-efficient battery systems, vital for the clean energy transition.

As research on battery mechanisms and thermal management advances, it has become evident to researchers that the internal temperature of a battery is the most crucial parameter in determining thermal runaway [7]. Electrochemical sensors enable macro-level battery temperature monitoring by measuring electrical parameters such as current [8], potential, and resistance [9]. Spinner *et al.* applied single-point electrochemical impedance spectroscopy (EIS) measurements at 300 Hz to successfully correlate the imaginary impedance with internal battery temperature, enabling real-time internal temperature monitoring [10]. In such circumstances, the internal temperature of the battery is considered to be homogeneously distributed. However, batteries generally operate with non-uniform temperatures due to locally enhanced surface exchange current density and sometimes develop localized-temperature hotspots [11], which can facilitate dendrite growth [12]. The advancement of optical sensors has further shifted battery diagnostics from average to localized monitoring [13]. The flexibility, small size, high-temperature resistance, electrochemical corrosion resistance, immunity to electromagnetic interference, and sensitivity to ambient temperature of optical fibers make them an excellent choice for identifying battery safety concerns [14]. Zhang *et al.* developed a multifunctional optical fiber sensor by using a Fiber Bragg Grating (FBG) for insertion into commercial 18 650 cells, enabling continuous internal monitoring of temperature and pressure during thermal runaway [15]. Wang *et al.* developed a ratio metric fluorescence optical fiber for real-time, *in situ* temperature monitoring of lithium-ion batteries, achieving a measurement accuracy of 0.12°C and demonstrating its effectiveness during charging and discharging cycles in a pouch cell [16]. Although these methods enable localization, temperature monitoring can only be performed in areas where the optical fiber has been specifically treated. Wang *et al.* developed an integrated functional electrode with optical fiber for *operando* temperature monitoring based on optical frequency-domain reflectometry (OFDR) during battery cycling without compromising electrochemical performance and demonstrated temperature distribution along the shape of the optical fiber [17]. However, since the number of detectable points remains low relative to the electrode surface area in current monitoring techniques, achieving precise *operando* distributed temperature monitoring across the entire lithium metal electrode during the whole battery life cycle remains unresolved.

Focusing on this conundrum, we have developed a system integrating physical sensing and super-resolution algorithmic approaches, achieving *operando* spatiotemporal super-resolution thermal monitoring (OST-SRTM) for LMBs. The OST-SRTM system provides the *in-situ*, super-resolution, spatiotemporal distributed thermal monitoring of LMBs for the first time. Based on Rayleigh backscattering (RBS), OFDR is a distributed fiber sensing technology with a spatial resolution up to several submillimeters [18]. We constructed an Archimedean spiral structure with single mode optical fiber (SMF) to transform a one-dimensional (1D) continuous point set into discrete points distributed across a two-dimensional (2D) plane. Then, we applied a super-resolution algorithm to achieve a precise spatial distribution resolution of 1820 points cm^−2^, and a temporal resolution of 1 frame per 3 seconds. During pouch cell cycling, we observed significant temperature variations and multiple hotspots in untreated cells, which indicated potential safety risks and ununiform lithium deposition. Several protection strategies were implemented, and our OST-SRTM system was used to verify the protection measures, together with battery monitoring. The lithium anode was patterned with pyramid imprinting and copper (Cu) mesh and formed an artificial solid electrolyte interface (SEI) layer by coating with polylactic acid (PLA). Instead of disassembling batteries after cycling to employ destructive techniques such as scanning electron microscopy (SEM), the effectiveness of these protection strategies for lithium anodes can be evaluated with high precision using the developed system. With the application of protection strategies and our developed system, we observed prolonged battery life, lower temperature rise, more uniform temperature distribution, and less occurrence of hotspots at the battery's capacity fading cycle. In all these lead to more uniform lithium deposition, a longer battery cycle lifetime, and reduced thermal runaway risk. This novel monitoring method greatly improves the safety and energy density of LMBs for sustainable energy applications by combining state-of-the-art optical technology with energy storage diagnostics and creating a strong framework for evaluating thermal management techniques.

## RESULTS

### Principle of the OST-SRTM system

**Figure 1.**
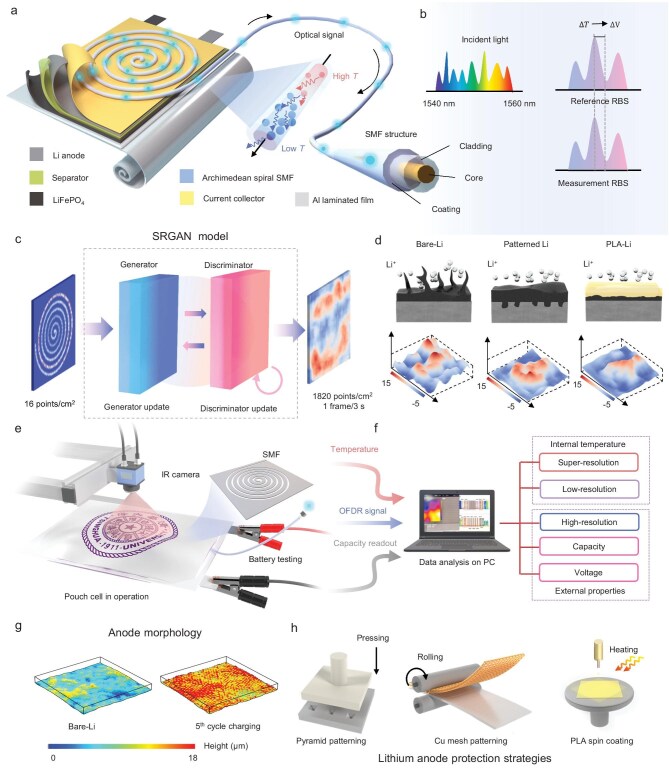
Principle and setups of the OST-SRTM system. (a) Configuration of the LMB pouch cell and the position of SMF in the cell. (b) Principles of RBS and 1D temperature-position along the fiber. (c) The temperature conversion from 1D to 2D and super-resolution algorithm application in temperature distribution. (d) Dendrite growth procedures of lithium anode (upper) and temperature maps of lithium electrodes after 50 cycles (left to right: Bare-Li, Patterned-Li, PLA-Li). (e) Experimental setups (battery testing system for capacity and capacity readout, IR camera for external temperature data acquisition, OFDR system for optical signal monitoring, laptop for all data acquisition and processing). (f) Relationship and functions of optical, electrical, and thermal signal processing. (g) Anode morphology simulation. (h) Lithium anode protection strategies: pyramid patterning, Cu mesh patterning, and PLA coating.

The Li/LiFePO_4_ (LFP) pouch cells were assembled by a straightforward method with Li anode, separator, and LFP cathode laminated together in the drying room. The Archimedean spiral SMF was bonded to the backside of the Cu current collector. Finally, the entire cell was encapsulated by aluminum-laminated film packages after injecting the electrolyte (Fig. 1a). OFDR achieved internal distributed temperature monitoring of the cell, which is composed of a tunable laser source (TLS), reference, and measurement arms (Fig. S1, Supporting information). The laser light from the TLS (1540–1560 nm) is split between these two arms. In the reference arm, the light serves as the local oscillator, while in the measurement arm, it is injected into a length of sensing fiber. When the temperature near the sensing fiber changes, a frequency shift occurs in the RBS between the measurement and reference light (Fig. 1b). Since the TLS is tuned linearly without mode hopping, the beat frequencies generated by the interference between the measurement and reference lights are directly proportional to the length of the sensing fiber [19]. By applying a Fourier transform to the beat frequencies, the signals in the optical frequency domain are converted into the spatial domain, allowing for the retrieval of the temperature distribution along different positions of the sensing fiber, which can be represented as:


(1) \begin{eqnarray*}
\frac{{\delta \lambda }}{\lambda } = \left( {\alpha + \xi } \right){\mathrm{\Delta }}T,
\end{eqnarray*}

where $\alpha $ and $\xi $ are the thermal expansion coefficient and thermo-optic coefficient of the optical fiber, respectively. To realize distributed temperature monitoring in 2D, we designed a spiral-shaped optical fiber structure. A mold was fabricated using 3D printing, with a groove (a line diameter of 1.2 mm) shaped in the form of an Archimedean spiral, which can be described as [20]:


(2) \begin{eqnarray*}
r = a + b\theta ,
\end{eqnarray*}

where $a = 0$, $b = \frac{{2.5}}{{10\pi }}$, *r* represents the radical distance from the center of the spiral to a point on the spiral, $\theta $ is the angular position around the origin. The SMF with a diameter of 0.9 mm was embedded into the prepared mold to maintain the shape and eliminate interference from stress (Fig. S2). By utilizing the parametric equation of the Archimedean spiral, the 1D position of the sensing fiber was mapped to the 2D coordinate distribution of the Archimedean spiral. To realize super-resolution, an algorithm based on a Super-Resolution Generative Adversarial Network (SRGAN) model was applied (Fig. 1c). We trained the model using a self-constructed dataset created from the OFDR system and infrared (IR) camera, combined with ceramic heating elements of various shapes. This approach enabled us to achieve super-resolution temperature mapping within battery interiors. There are two main strategies for surface pretreatment to stabilize the lithium anode in order to reduce hotspots and slow down the battery's aging process: patterning and artificial solid electrolyte interface [21]. We employed pyramid imprinting, Cu mesh, and coating with PLA to protect the lithium anode (Fig. 1d and Fig. S3).

### Signal acquisition and processing of the cell

In order to investigate the temperature distribution and uneven lithium deposition within the pouch cells, the experimental setups for signal acquisition and processing of the cell are shown in Fig. 1e and f. To achieve spatiotemporal temperature monitoring, we placed the coiled optical fiber and mold into the LMB, which was cycled continuously until a significant capacity drop was reached. During the cycling process, the cell was connected to a battery testing system in order to verify the insertion of the optical fiber shows no negative impact on the battery performances [22]. As shown in Fig. S2d, embedding optical fibers show no impact on internal resistance, thermal conductivity, and overall electrochemical behavior of the cell. One end of the optical fiber was connected to the OFDR testing system to monitor internal temperature, while the external temperature was monitored by an IR camera. To investigate the relationship between heat generation and uneven lithium deposition during normal operation conditions, the Bare-Li anode morphology was simulated. During the charging process of LMBs, lithium-ions migrate from the electrolyte to the surface of the anode, where they deposit on the lithium metal side. This deposition process is influenced by various factors, such as the lithium-ion concentration gradient and the tip effect, leading to uneven lithium deposition on the lithium metal surface. According to simulation results, after five charge-discharge cycles, the morphology of pure lithium deposits becomes noticeably rougher, with a significant reduction in density and compactness, and a marked increase in overall deposition layer thickness (Fig. 1g). This uneven deposition not only creates unfavorable conditions for the subsequent formation of a thicker ‘dead lithium’ layer and dendrite growth but also increases the internal resistance of the battery, thereby impacting its cycle life and safety. During the discharge process, although the overall thickness of the anode decreases, some deposited lithium remains on the anode surface due to limitations in charge-discharge efficiency (Fig. S4). These residual lithium deposits further exacerbate the uneven deposition phenomenon, promoting the accumulation and extrusion of ‘dead lithium’. This process leads to increasingly dense lithium deposits in localized areas, contributing to heat accumulation during charge-discharge cycles and posing challenges to battery thermal management and long-term stability. This series of issues severely affects the overall performance and lifespan of the battery [23]. To promote the uniformity of Li^+^ deposition, three lithium anode protection strategies were provided (Fig. 1h). The first two are based on surface patterning [24]: (1) pressing a pyramid-shaped mold into the lithium foil and (2) rolling a copper mesh to create micro patterns. Patterning directed lithium dendrite growth into the grooves, thus minimizing the formation of hotspots. The third strategy was coating the lithium foil with PLA, leading to more uniform lithium-ion deposition and improved battery performance by constructing a built-in electric field [25]. The OST-SRTM system was applied for more precise spatiotemporal monitoring for different lithium anodes.

### Super-resolution of temperature mapping

Although the OFDR system can provide temperature variations along the fiber, and the temperature information can be converted from 1D to 2D by coiling, there are still monitoring gaps between the fibers. Applying regression analysis [26], interpolation methods [27], and Bayesian inference [28] can fill in the missing data. However, these traditional interpolation methods tend to result in detail loss or blurring. In recent years, machine-learning-based super-resolution techniques have been applied to convert low-resolution images into higher-resolution ones while reconstructing details. Since there are no other precise methods for measuring internal battery temperatures, we launched external temperature experiments by attaching ceramic heating elements of different shapes to optical fibers arranged in an Archimedean spiral. At the same time, an IR camera was used to collect data to construct the training dataset, this method is widely recognized for studying similar thermal behavior phenomena. Using this dataset, we applied machine learning to achieve super-resolution for temperature mapping, filling in the gaps where fibers were absent.

**Figure 2.**
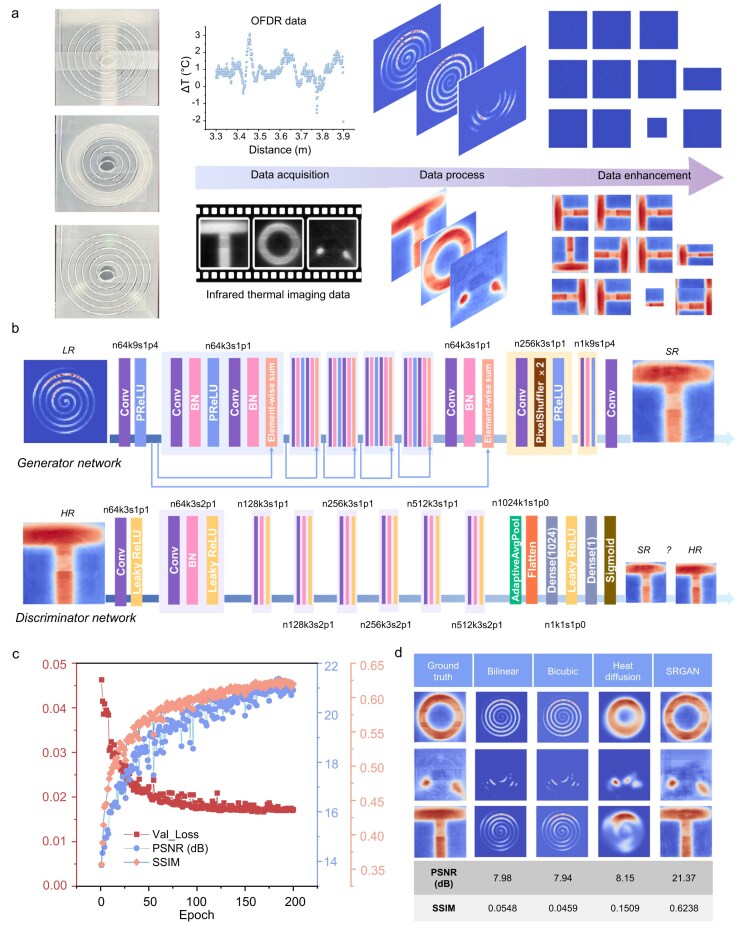
Super-resolution based on SRGAN applied to OFDR temperature data. (a) The dataset constructed by OFDR and infrared thermal imaging. (b) The structure of the SRGAN model. (c) Changes in validation loss, PSNR, and SSIM during training process. (d) Comparison of different super-resolution reconstruction methods applied to thermal field images.

As shown in Fig. 2a, T-shaped, circular, and two-point ceramic heating elements were placed beneath the SMF. The temperatures of the elements were controlled by a power supply. Both the OFDR system and the IR camera were used simultaneously to record the temperature data. The OFDR system captured the data in 1D format, which was then processed to form the Archimedean spiral. Areas without temperature readings were filled with room temperature values (22°C). Meanwhile, the IR camera recorded videos of the same region's temperature changes. The videos were framed to images and synchronized with the OFDR system's data collection time, forming the raw dataset. To avoid overfitting due to the limited dataset diversity, we employed data augmentation techniques. Images from the raw dataset were transformed using random rotations, reflections, translations, scaling, brightness and contrast adjustments, Gaussian blur, noise addition, and cropping. These modifications generated a more diverse and robust dataset for training. The network was constructed based on the SRGAN model, as shown in Fig. 2b. The generator network was a deep network consisting of 5 ResNet blocks, designed to enhance information flow across layers using skip connections and to prevent gradient vanishing as the network depth increases. For example, ‘n64k9s1p4’ refers to 64 convolutional kernels of size 9 × 9, with a stride of 1 and padding of 4. The discriminator network is a CNN, where Leaky ReLU (α = 0.2) is used as the activation function to avoid the issue of dead neurons from negative outputs. At the end of the network, a dense block is followed by a sigmoid function to perform binary classification, scoring the high-resolution (HR) and super-resolution (SR) images. In our optimal model, we achieved a Peak Signal-to-Noise Ratio (PSNR) of 21 dB and a Structural Similarity Index Measure (SSIM) of 0.62 on the test set (Fig. 2c), successfully transforming the 1D temperature data from the OFDR system into super-resolution 2D temperature maps. Specifically, we evaluated input images of sizes 32 × 32, 64 × 64, and 128 × 128, while adjusting only the final upsampling factor in the model to ensure proper functionality (Table S3) of Mean Absolute Error (MAE), and Mean Squared Error (MSE). Figure 2d shows the comparison results of different heating elements based on SRGAN and several traditional methods, including quadratic interpolation, cubic interpolation, heat diffusion based on Gaussian filtering with a diffusion coefficient of 0.5 over 200 iterations (based on bicubic interpolation). In terms of accuracy, SRGAN demonstrated a significant performance advantage over all other methods. The spatial resolution of our developed system is 1820 points cm^−2^, and the temporal resolution is 1 frame per 3 seconds.

### Performance of the OST-SRTM system

**Figure 3.**
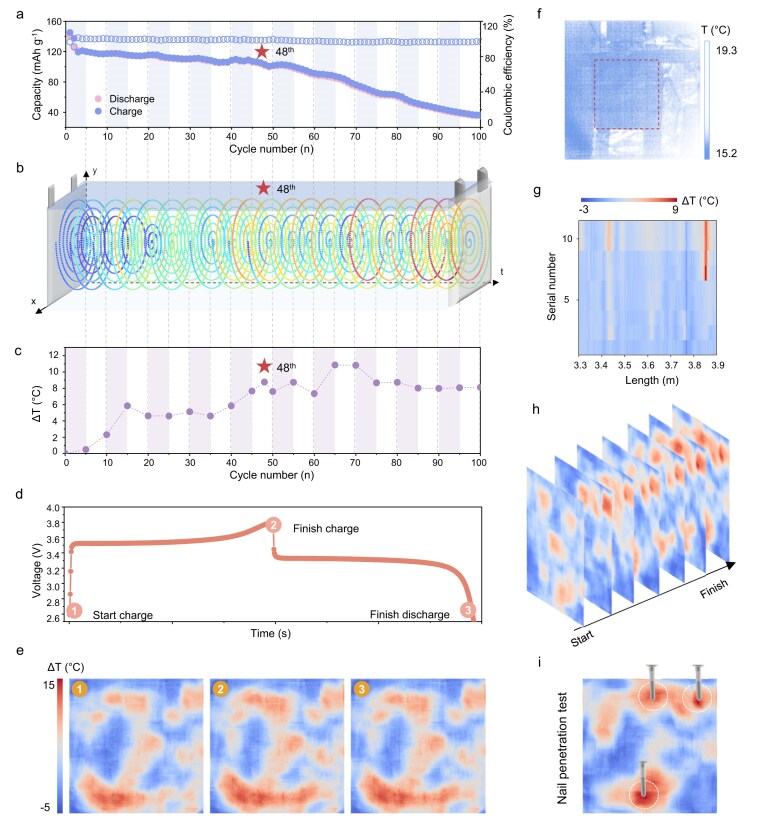
Electrochemical properties and spatiotemporal temperature distribution over the entire lifecycle and nail penetration test of the LMB. (a) The capacity curve over 100 cycles. (b) 3D spatiotemporal temperature distribution during cycling. (c) The average temperature of the cell during 100 cycles. (d) The cyclic voltammetry curve of the battery at the 48th cycle (① start charge, ② finish charge, and ③ finish discharge). (e) Super-resolution temperature map at the start of the 48th cycle, after charging, and after discharging. (f) The actual temperature before the start of the penetration test taken by an IR camera. (g) The heatmap of 1D fiber temperature variation over time. (h) Time distribution super-resolution temperature map during the nail penetration test. (i) Nail penetration locations and hotspots.

As shown in Fig. 3, an untreated LMB pouch cell was cycled for 100 times at 0.5 C to realize temperature and electrochemical monitoring throughout its lifecycle. A capacity degradation (80% of the original capacity) was observed by the 48th cycle (Fig. 3a). By monitoring the spatiotemporal temperature over the entire lifecycle of the cell, the temperature evolution inside the cell is effectively calculated. The three-dimensional (3D) spatiotemporal temperature distribution was mapped according to the shape of the SMF, which entered the pouch cell from the negative terminal (Fig. 3b). It is suggested that a maximum temperature gradient of <5°C is the optimal temperature range for Li batteries, which achieves a suitable balance between performance, battery life, and safety [29]. During the period when the capacity remained stable, no significant heat accumulation was observed; the average temperature increase remained below 5°C, with a relatively uniform temperature distribution (Fig. 3c). This aligns with the balance between heating and cooling processes within the cell. However, once capacity decline began, numerous hotspots were detected. By the 100th cycle, heat accumulation persisted, accompanied by a deterioration in the cell's heat dissipation capacity, which further contributed to capacity decline.

A detailed analysis of the cell at its 48th cycle was conducted (Fig. 3d and e). During charging, the internal temperature of the cell gradually increased. Across the entire cell, the most prominent hotspot exhibited a maximum temperature rise of 15.1°C from the start of the first cycle, while the minimum temperature increase was 3.9°C, resulting in an 11.2°C temperature differential. The current passing through the cell generates Joule heat. Additionally, the migration kinetics of Li^+^ and the deposition process involve the conversion of chemical energy into thermal energy, leading to some degree of heat accumulation. Furthermore, side reactions, such as electrolyte decomposition may occur under high charging voltages, producing excess heat and causing significant temperature variations during the charging cycle. After discharge, the overall cell temperature decreased by 1.8°C (with a maximum temperature rise of 13.3°C and a minimum temperature increase of 2.8°C). During discharging, apart from Joule heating, Li^+^ migration and the lithiation process at the cathode also contributed to heat generation. Continued cycling exacerbates uneven dendrite deposition on the anode, creating localized high-resistance regions and leading to uneven heat accumulation. In severe cases, excessive dendrite growth may trigger micro–short circuits, causing a rapid increase in temperature and a significant risk of organic electrolyte combustion. In conclusion, monitoring the temperature changes of the cell during the charging and discharging processes is crucial for battery safety.

A nail penetration test was initiated to verify battery performance upon severe external force (Fig. 3f–i). A steel nail with a diameter of 5 mm was stapled with a constant pressure of 13 MPa into the pouch cell. This penetration was carried out during the discharge phase of the cell, specifically at a rate of 0.5 C, to simulate conditions relevant to thermal runaway scenarios and to better understand the thermal response during such high-stress mechanical events. Figure 3f shows the surface temperature before penetration (15.2–19.3°C) taken by an IR camera, and the section enclosed by the red dashed line represents the cell. In this test, we performed pinning at three points on the cell. Figure 3g illustrates the heatmap of the cell over time. When pinning occurs, the temperature at the points rose rapidly and remained elevated. The super-resolution temperature distribution is shown in Fig. 3h and i. The circled points represent the pinning locations, and the temperature sharply increases to 9.9°C, 4.7°C, and 5.5°C.

### Application of the OST-SRTM system

**Figure 4.**
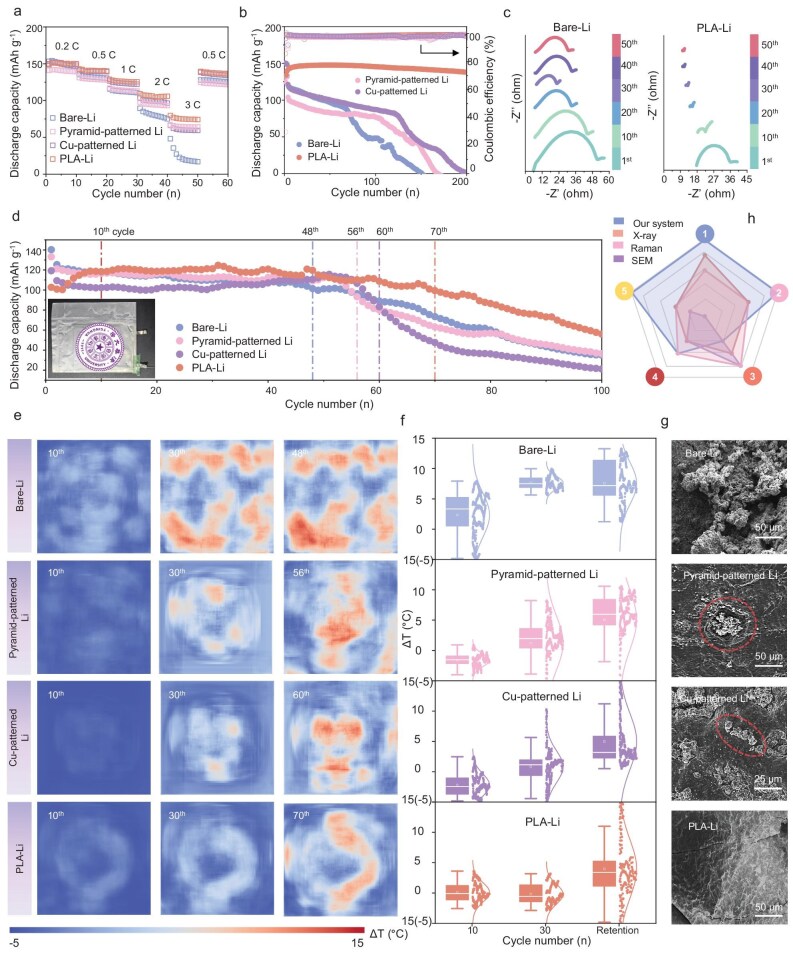
Validation of the effectiveness of lithium anode protection strategies. (a) Rate performance, (b) cycling performance of button cells with Bare-Li, Pyramid-patterned Li, Cu-patterned Li, and PLA-Li from 1 to 5 mA cm^−2^. (c) Resistance of Bare-Li and PLA-Li under different cycle numbers. (d) Cycling performance of pouch cells with Bare-Li, Pyramid-patterned Li, Cu-patterned Li, and PLA-Li. (e and f) Super-resolution images and box plots of temperature variations of different pouch cells at 10th cycle, 30th cycle and the retention cycle (48th, 56th, 60th, 70th cycles, respectively). (g) SEM images of Bare-Li, Pyramid-patterned Li, Cu-patterned Li, and PLA-Li (from up to down) after 50 cycles. (h) Radar chart of different monitoring methods (① Real-time, ② Non-destructive, ③ Precision, ④ Equipment volume, ⑤ Measurement area).

During cycling, uncontrolled dendrite growth during electrodeposition can accelerate thermal accumulation [30,31]. Therefore, stabilizing the lithium anode via interface engineering is a potential way to reduce hotspots and slow down battery aging. The OST-SRTM system was used to validate protection methods. We first used button cells to verify the improvement in LMB performance achieved through lithium anode protection. As shown in Fig. 4a, the rate performance of PLA-Li is significantly better than that of Pyramid-patterned Li and Cu-patterned Li, with Bare-Li demonstrating the worst performance. This clearly demonstrates the benefits of different protection strategies for discharge capacity at high current densities. To further assess the impact of these protection methods on extending battery cycle life, cycling stability tests were conducted (Fig. 4b). PLA-Li exhibited the highest stability, maintaining a capacity retention rate of 99.1% after 200 cycles at a current density of 1 C, with a per-cycle capacity decay rate of only 0.0045%. This strongly confirms the effectiveness of PLA as an artificial SEI. PLA's lithium carboxylate formation improves ion transport during charge-discharge cycles, while its piezoelectric effect establishes a sensitive, responsive, strength-adaptive electric field that further promotes Li⁺ migration and uniform lithium deposition, thereby extending battery cycle life. In comparison, Pyramid-patterned and Cu-patterned Li cells retained 80% of their initial capacity after 102 cycles and 108 cycles, representing extensions of 47.8% and 56.5% over Bare-Li, which was retained after 69 cycles, indicating these methods also contribute positively to battery stability. Post-cycling analyses of the anode interfaces revealed substantial differences across electrode types. EIS was used to measure the resistance values of these electrodes, with the corresponding results presented in Fig. 4c and Fig. S5. For Bare-Li, post-cycling measurements showed high charge transfer resistance and interfacial resistance, leading to significant Joule heating during subsequent charge-discharge processes. This heat accumulation accelerates the degradation of internal chemical components, ultimately shortening the battery's cycle life. In contrast, anodes with protective strategies showed significantly reduced resistance values after the same number of cycles. The PLA-Li anode, in particular, demonstrated superior interfacial stability and charge transfer efficiency, effectively reducing cell degradation and heat buildup and thus substantially extending the LMB lifecycle.

**Table 1.** Non-destructive monitoring methods for batteries.


Type	Sensing system	Location	Monitoring indicators	Dimension	Resolution	Ref.

Electrical	EIS	External	Impedance	Single point	/	[38]
	Battery management system (BMS)	External	Voltage, current	Single point	/	[39]
	Thermal-wave sensor	Surface	Thermal conductivity	Single point	/	[40]
Acoustic	Ultrasonic imaging	Surface	Ultrasonic transmittance	Single point (scan to 2D)	Spatial: 250 points cm^−2^ Temporal: 1 frame per 0.5 hours	[41]
Optical	Chirped-FBG	Internal	Temperature	Single point (scan to 1D)	Spatial: 347 points cm^−1^ Temporal: 1 point per 55 milliseconds	[42]
	Optical FBG sensor	Internal	Temperature, strain	Single point	/	[43]
	OFDR	Internal	Temperature, strain	2D	Spatial: 9 points cm^−2^ Temporal: 1 frame per 1 cycle (4 hours)	[17]
	OFDR	Internal	Strain	2D	/	[44]
	OST-SRTM	Internal	Temperature	2D	Spatial: 16 points cm^−2^ (super-resolution to 1820 points cm^−2^) Temporal: 1 frame per 3 seconds	This work

Then, pouch cells were fabricated to verify the impact of the anode protection strategies on ununiform lithium deposition and hotspot formation. To reduce the testing duration while continuously monitoring the temperature changes of pouch cells throughout the entire cycling process, a straightforward approach was adopted. High-loading commercial LiFePO₄ (loading: 12.73 mg cm⁻²) was used as the cathode, paired with different lithium metal anodes, to evaluate the protection and monitor temperature variations throughout the entire cycling process. Based on the cycling stability and capacity retention rates of different pouch cells, PLA-Li demonstrated the best electrochemical performance. The response spectra of SMF to temperature were also tested (Fig. S6) to observe the overall temperature variation trend inside the battery, with electrochemical and temperature properties assessed concurrently. The pouch cell with Bare-Li experienced capacity degradation (80% of the initial capacity) at the 48th cycle, while the cells treated with the Pyramid-patterned, Cu-patterned, and PLA showed degradation at the 56th, 60th, and 70th cycles with delayed capacity degradation (16.7%, 25%, and 45.8% longer), respectively (Fig. 4d). The spatiotemporal temperature maps of four cells were measured by our designed system (Fig. 4e and f). Over the first 10 cycles, the capacity and performance of batteries remained stable, with no significant hotspots observed and an average temperature below 2.3°C across all four cells. Box plots combined with scatter density plots illustrated the central tendency and dispersion of temperature distributions for each electrode type after cycling. Bare-Li exhibited the widest temperature range (from −5°C to 8°C) and a relatively high median, indicating significant temperature increases during cycling. Protected anodes showed more concentrated temperature distributions, with narrower ranges, suggesting that the protection strategies can partially suppress temperature variations. As cycling progressed, heat accumulation became evident. Bare-Li experienced an obvious temperature rise, with a clear upward trend indicating susceptibility to heat accumulation over multiple cycles. Pyramid-patterned and Cu-patterned Li maintained more stable temperatures with slight increases, and PLA-Li exhibited the smallest temperature rise and the most stable distribution. At capacity retention cycles, anodes with protection displayed lower average temperature rises (3.1°C, 2.6°C, and 0.7°C) compared to Bare-Li (5.3°C), and fewer hotspot areas at later capacity degradation cycles. Scatter density plots for Bare-Li showed considerable temperature fluctuations, indicating an uneven distribution with multiple hotspots. PLA-Li demonstrated the best thermal management capability, making it suitable for applications requiring high thermal stability. Cu-patterned Li follows closely, with Pyramid-patterned Li providing moderate stability. These observations indicate an interaction between hotspots and capacity degradation. After 100 cycles, cells that retained greater capacity showed more heat accumulation; ultimately, the temperature of all the batteries tends to converge. This suggests that as battery capacity degrades, remaining capacity continues to influence internal temperature under normal cycling conditions (Figs S7–S10).

To further validate trends in temperature changes and hotspot distribution, anodes with different protection strategies were observed by SEM before cycling (Fig. S4) and after 50 cycles (Fig. 4g). The Bare-Li surface exhibited highly uneven lithium deposition and a notable increase in thickness, leading to elevated interfacial resistance during cycling. In contrast, the deposited lithium preferentially grew in the patterned areas, especially in the holes of Pyramid-patterned and Cu-patterned Li, which improves the uniformity on the electrode and prevents uneven deposition and heat accumulation. As for PLA-Li, the artificial SEI layer with piezoelectric effects and high ionic conductivity facilitated ion transport and uniform deposition beneath the SEI. After cycling, the lithium deposition on the anode side remains remarkably uniform, which is more conducive to the extension of the battery's cycle life [25]. Traditional methods for assessing battery health and conducting 2D analyses of Li^+^ deposition, such as X-ray [32,33], Raman spectroscopy [34,35], SEM [36] provide valuable insights but often lack real-time, non-destructive capabilities across large areas. In comparison, our developed OST-SRTM system enables precise, real-time monitoring across the entire cell area without compromising battery integrity, offering a superior diagnostic tool in terms of accuracy, coverage, equipment size, and non-destructive (Fig. 4h).

Non-destructive methods have been developed to monitor the status of batteries as shown in Table 1, which can be categorized into electrical, acoustic, and optical. Most electrical monitoring methods focus on single point and external indicators such as impedance, voltage, current, and thermal conductivity. Acoustic methods transform a single point into a 2D dimensional representation through scanning. Optical methods have emerged as the most promising technology for monitoring distributed temperature and strain variations inside batteries, owing to their advantages of electromagnetic interference resistance, high sensitivity, corrosion resistance, and multi-point monitoring capabilities [37]. Compared to other optical sensors, our developed OST-SRTM system offers advantages such as *operando* observation, 2D spatiotemporal (1 frame per 3 seconds), and high-resolution (16 points cm^−2^, super-resolution to 1820 points cm^−2^), as summarized in the table below.

## CONCLUSION

This study introduces a first OST-SRTM system for LMBs that enables *operando* monitoring of temperature distribution across the lithium anode, achieving an initial resolution of 16 points cm^−2^ via OFDR, and super-resolution to 1820 points cm^−2^, with a temporal resolution of 1 frame per 3 seconds. The developed system provides real-time, non-destructive monitoring over a large measurement area with high precision. Our findings reveal that the presence of localized hotspots can precipitate thermal runaway and affect lithium deposition. The implementation of protective strategies, including pyramid, Cu mesh patterning, and PLA significantly enhances temperature uniformity and extends battery lifetime. Cells with these treatments exhibit capacity degradation delays of 16.7%, 25%, and 45.8%, and average temperatures consistently lower than untreated cells by 4.02°C, 4.32°C, and 6.84°C, respectively. Monitoring with OST-SRTM reveals that the application of these strategies has reduced the occurrence of hotspots during capacity fade. These findings highlight the critical need for precise thermal management in LMBs, as even minor temperature variations can lead to substantial performance impacts. The OST-SRTM system demonstrates general applicability across different battery technologies, such as sodium-ion and sodium-metal batteries, with potential for direct implementation without modification due to the similarities in the physicochemical properties of sodium and lithium. Future research could explore further enhancements in thermal stability and the applicability of OST-SRTM across various battery technologies, such as solid-state batteries, with adaptations required for solid-state batteries due to the impact of external pressure on sensing signals, which is being explored in ongoing research. This work not only advances battery diagnostics but also lays the groundwork for safer and more efficient energy storage solutions, which is critical for sustainable energy systems.

## METHODS

### Lithium anode protection strategies

Untreated lithium anode is named as Bare-Li.

1. Pyramid-patterned Li
  A pyramid mold was fabricated through high-precision 3D printing using the BMF nanoArch S140. The mold (50 mm × 50 mm × 0.5 mm) was characterized by a height (h) of 250 μm, a width (w) of 250 μm, and a spacing (l) between pyramids of 200 μm. A pressure of 2 MPa was applied, embedding a micro structured pattern into the surface of the lithium, named Pyramid-patterned Li (Fig. S4a).
2. Cu-patterned Li
  A pressure of 1 MPa was exerted onto the lithium surface with a 250-mesh copper grid. The imprinting was performed initially in a direct orientation, followed by a second imprinting after rotating the grid by 45 degrees (Fig. S4b), named Cu-patterned Li.
3. PLA-Li
  A moderate amount of PLA was dissolved in DMSO solution, stirred at 130°C for 5 h to dissolve followed by continuous stirring at 80°C for 24 h. PLA solutions with concentration of 1.2% (mass ratio) were scraped onto the lithium metal surface, named PLA-Li (Fig. S4c) [25].

### Fabrication of lithium metal pouch cells with SMF

The Li/LFP pouch cells were assembled in the drying room. The Bare-Li, Pyramid-patterned Li, Cu-patterned Li, or PLA-Li, separator, and LFP cathode were laminated together. Then the SMF with mold was bonded to the backside of the copper current collector. Finally, the entire cell was encapsulated by Al-plastic film packages after injecting the electrolyte. The electrolyte was 1.0 M LiPF_6_ in EC/DMC/DEC (1: 1: 1, v:v).

### Optical test

The OFDR system analyzer was provided by MegaSense (Wuhan, China), with a spatial resolution of 1 mm, and a temperature resolution of 0.1°C. One end of the optical fiber extends out from the battery and is connected to the OFDR system, while the other end is coiled into the mold.

### Electrochemical test

All button cells of 2032-type were assembled in an argon-filled glove box with H_2_O and O_2_ level below 0.01 ppm for electrochemical measurements. Li/Li symmetric cells were assembled using two Li foil electrodes (Bare-Li, PVDF-Li, or PLA-Li); 1 M LiTFSI in 1,3-dioxolane/1,2-dimethoxyethane (DOL:DME = 1:1 in volume) with 2 wt% lithium nitrate (LiNO_3_) additive was used as the electrolyte for the symmetric and half cells. The Li-LFP cells were assembled in a glove box with LiFePO_4_ as cathode and different Li metal foils as anodes. The mass loading of the LFP was ∼10.52 mg cm^−2^. The electrolyte was 1.0 M LiPF_6_ in ethylene carbonate/dimethyl carbonate/diethyl carbonate (EC:DMC:DEC = 1:1:1 in volume). The rate performance and cycling performance of full cells and pouch cells were tested by electrochemical workstation (LAND dtx64) at different current densities.

### Postmortem of super resolution

The dataset was collected using the OFDR system and infrared thermal imaging (X640F). Data processing and machine learning tasks were performed on a CPU (Intel i5-14600KF) and GPU (NVIDIA GeForce RTX4060 Ti 16 G). The SRGAN machine learning model was implemented using PyTorch.

## Supplementary Material

nwaf088_Supplemental_File

## Data Availability

The data supporting the findings of this study are available from the corresponding author on reasonable request.

## CODE AVAILABILITY

The related code and dataset have been open-sourced on GitHub. https://github.com/ZecongLIU/Super-resolution-for-OFDR-in-battery.git
