# Supplementary material for: Operando spatiotemporal super-resolution of thermal events monitoring in lithium metal batteries
Source: Natl Sci Rev. 2025 Mar 6;12(5):nwaf088. doi: 10.1093/nsr/nwaf088 (PMC11987594; doi:10.1093/nsr/nwaf088)
Supplement: nwaf088_Supplemental_File [file nwaf088_supplemental_file.zip › Supplementary data.pdf]

Supplementary Information

Operando Spatiotemporal Super-Resolution of Thermal Events Monitoring in Lithium Metal Battery

Author Information

Affiliations

[1] Tsinghua Shenzhen International Graduate School, Tsinghua University, Shenzhen, Guangdong, 518055, China.

[2] College of Materials Science and Engineering, Shenzhen University, Shenzhen, Guangdong, 518060, China.

[3] School of Physics, Northwest University, Xi'an, Shaanxi, 710127, China.

Chonghao Zhang[1#], Zecong Liu[1#], Zhoujie Lao[1], Yuting Zhou[1], Xiao Xiao, Tao Feng[1], Ruohui Wang[3], Chengshuai Chang[1,2*], Guangmin Zhou[1*], and Xun Guan[1*]

Contributions

[#]These authors contributed equally to this work.

Corresponding author

Correspondence to: Chengshuai Chang, Guangmin Zhou, and Xun Guan.

cschang@szu.edu.cn; guangminzhou@sz.tsinghua.edu.cn; xun.guan@sz.tsinghua.edu.cn.

Basic principle and configuration of OFDR

By analyzing the intensity variation of the Rayleigh backscattered light, the attenuation information along the SMF can be obtained. When the external temperature changes, the Rayleigh scattering spectrum shifts accordingly, with the magnitude of the shift being directly related to the temperature.

The establishment of an Optical Frequency Domain Reflectometry (OFDR) system primarily involves components such as a linearly tunable laser (TLS), an interferometer structure (reference and measurement arms), a photodetector (PD), and a signal processing unit (DAQ) (Fig. S1a). The coherent light emitted by the linear swept-frequency laser enters a fiber-based Mach-Zehnder interferometer through a coupler[1,2]. Assuming that the light source frequency changes linearly over time, its instantaneous frequency can be represented as[3]:

$$f(t) = f_0 + kt\#(1)$$

, where f_0 and k represent the initial frequency of the light source and the sweep rate, respectively. One part of the light travels directly along the reference path to the coupler, serving as the reference light. The other portion enters the sensing fiber through a circulator. The optical fields of the reference light (without delay) $E_1(t)$ and the signal light (with delay) $E_2(t)$ reflected by the two arms of the Michelson interferometer can be represented as (assuming the reflectance R = 1):

$$E_1(t) = E_0 \exp\{j(\omega_0 t + \pi k t^2)\}\#(2)$$

$$E_1(t) = E_0 \exp\{j[(\omega_0 (t - \tau_0) + \pi k (t - \tau_0)^2)\}\#(3)$$

, where E_0 represents the amplitude of the reference light, $\omega_0 = 2\pi f_0$, $\tau_0 = 2nx_0/c$, $2nx_0$ is the optical path difference between the two arms of the interferometer, and c is the speed of light in vacuum. As light propagates through the sensing fiber, Rayleigh scattering signals are continuously generated and return to the system. These backscattered Rayleigh signals serve as the signal light. Because the conditions for coherence are satisfied, the signal light reaches the coupler via the circulator and interferes with the reference light. The reference light and the signal light interfere within the coupler, resulting in a beat signal I(t), which can be represented as:

$$I(t) = |E_1(t) + E_2(t)|^2 = 2E^2(1 + \cos(\omega_b t + \theta_c))\#(4)$$

, where $\omega_b = 2\pi k \tau_0$ represents the beat signal frequency and θ_c is a constant phase term. By neglecting the DC component and the constant phase term, it can be concluded that the beat signal frequency is proportional to the difference in the interferometer arm lengths. This interference is then converted into an electrical signal by a photodetector for subsequent analysis and demodulation.

During the linear frequency sweep of the laser, scattering points at different positions along the fiber correspond to different frequencies. By analyzing the spectrum's frequency, the location of scattering points along the sensing fiber can be determined, thus enabling fiber positioning. Moreover, OFDR technology also facilitates distance measurement. By applying a Fourier transform to convert the OFDR raw signal to the frequency domain, a sliding window is used to select Rayleigh scattering signals from different positions, with a window size of:

$$\Delta X = N\Delta x \#(5)$$

, where $\Delta x = c/2n\Delta F$, is the spatial resolution of the OFDR system, the spatial resolution of the temperature measurement is ΔX. Next, the Rayleigh scattering signals within the sliding window are transformed into the wavelength domain through an inverse Fourier transform. Finally, the reference signal and the measured signal are cross-correlated in the wavelength domain. At this stage, the wavelength shift of the correlation peak reflects the temperature information at the position of the sliding window on the optical fiber. By changing the sliding window position and repeating the inverse Fourier transform and cross-correlation steps, distributed temperature measurement can be achieved. When the temperature around the optical fiber changes, the wavelength shift produced by the Rayleigh scattering pattern can be represented as:

$$\frac{\delta\lambda}{\lambda} = (\alpha + \xi)\Delta T \#(6)$$

, where α and ξ are the thermal expansion coefficient and thermo-optic coefficient of the optical fiber, respectively. The location of scattering points within the fiber can be inferred based on the spectral frequency[2]. The frequency difference corresponds to the distance between two scattering points (Fig. S1b-d). The optical frequency resolution in an OFDR system is defined as the minimal measurable optical frequency shift. The relationship between the measurand resolution and the effective sensing spatial resolution can be obtained as

$$\delta S_{min} = \frac{c}{2n_g \Delta X \times RES} \#(7)$$

where RES, as an intrinsic characteristic of fiber, is defined as the ratio of the RBS shift to the variation in the measurand. Here, n_g denotes the group refractive index. The temperature resolution is

determined from the frequency shift, exhibiting a linear relationship with a coefficient of approximately 0.66 GHz/°C. In this study, the temperature resolution is set to 0.1 °C, a limitation imposed by the system's hardware, making further improvements challenging. However, this resolution is sufficient to meet the requirements of practical applications. Consequently, the primary focus of this research is on enhancing spatial resolution.

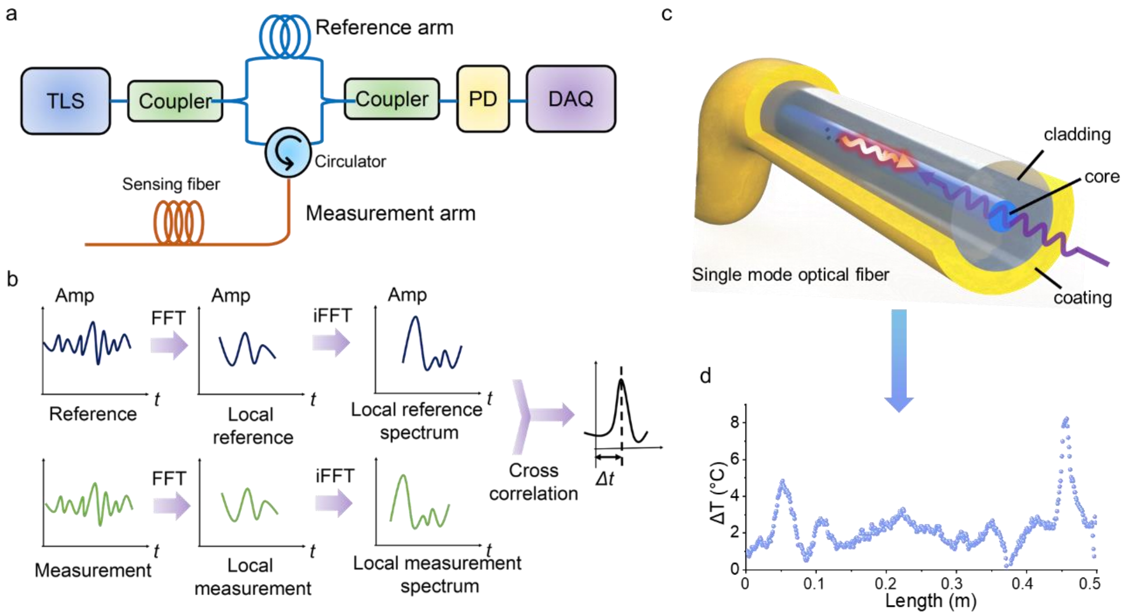

Fig. S1 | a OFDR system configuration. **b** Demodulation procedure of RBS shifts. **c** RBS shifts in SMF. **d** Temperature-length variation along the fiber.

Archimedean spiral with single mode fiber (SMF)

The Archimedean mold was designed using SOLIDWORKS software and subsequently fabricated through 3D printing, utilizing a Creality Print K1 Max printer. Given that battery operation necessitates compression through the use of a fixture, a single-mode fiber (SMF) was embedded within the mold to ensure the fiber's position remained stable during the entire battery operation. This embedding allows for consistent operando monitoring of the system without positional discrepancies. To minimize any interference that could arise from mechanical stress during battery compression, a pressure-relief space of 0.3 mm was incorporated into the mold. This design feature effectively

mitigates the impact of stress interference, thereby preserving the integrity of the temperature or strain measurements conducted via the embedded fiber. Furthermore, to eliminate optical artifacts such as Fresnel reflections at the fiber’s terminal end, a knot was tied at the fiber's end with a radius of 5 mm, as depicted in Fig. S2a. The mold, containing the embedded SMF, was carefully positioned on the external surface of the lithium anode current collector, using a green adhesive to maintain its placement (as illustrated in Fig. S2b). This assembly was then encapsulated with an aluminum-laminated film to provide additional protection, as shown in Fig. S2c. The combination of these steps ensured both stability and reliable operando data collection throughout the battery's operational cycle. As shown in Fig. S2d, embedding optical fibers show no impact on overall electrochemical behavior of the cell.

The Archimedean spiral emerges as an optimal design choice. This geometric structure establishes a straightforward mathematical relationship between the radial distance and the polar angle across all directions. A key advantage of this configuration is that shorter fiber segments can uniformly cover the same area while being exclusively designated for sensing. Furthermore, due to its ability to detect temperature variations at all angles within the entire 2D plane, the temperature between two adjacent loops can be easily determined. By leveraging the mathematical properties of the Archimedean spiral, it is possible to precisely map the 1D location of the sensing fiber to its corresponding 2D distribution within the polar coordinate system.

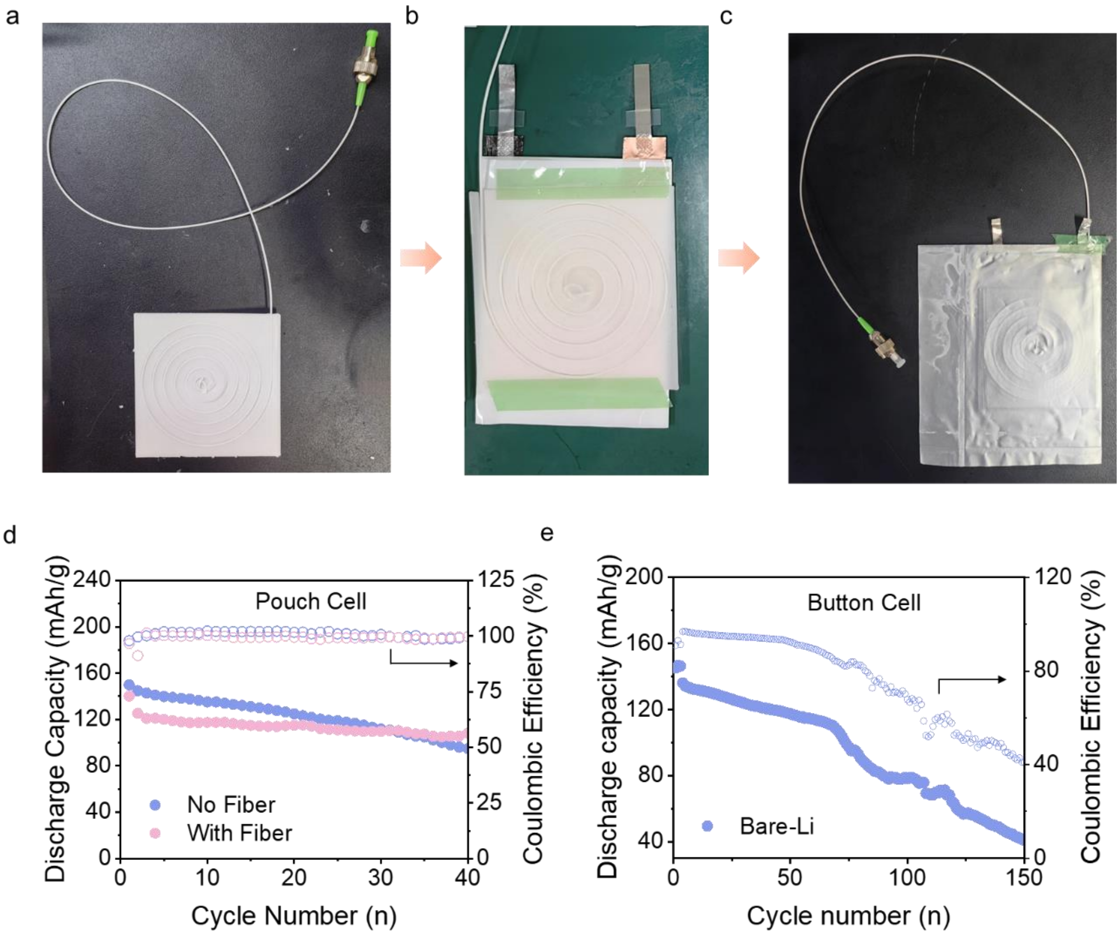

Fig. S2 | a The Archimedean mold with SMF. **b** Position of the mold with SMF. **c** Pouch cell. **d** Discharge capacity of Bare-Li pouch cells with and without SMF. **e** Discharge capacity and coulombic efficiency of Bare-Li button cell.

Protection strategies for lithium anode

In our research, we employed three distinct protection strategies to enhance the performance and stability of the lithium anode. The first strategy utilized a pyramid mold, which was fabricated through high-precision 3D printing using the BMF nanoArch S140 printer. The mold was designed with dimensions of 50 mm × 50 mm × 0.5 mm, and the individual pyramids on the mold were characterized by a height (h) of 250 μm, a width (w) of 250 μm, and a spacing (l) between pyramids of 200 μm. To imprint the pyramid structure onto the lithium anode, a force of 2 MPa was applied,

thereby embedding a microstructured pattern into the surface of the lithium, as depicted in Fig. S3a. This microstructuring aimed to enhance surface uniformity and reduce localized current densities[4], which are known to contribute to hotspot formation and dendritic growth. The second strategy involved imprinting using a commercially available 250-mesh copper grid[5,6] (Fig. S3b). The imprinting process was conducted by applying a pressure of 1 MPa to the lithium surface. To improve the uniformity of the surface modifications, the imprinting was performed twice: initially in a direct orientation, followed by a second imprinting after rotating the grid by 45 degrees. This dual-stage imprinting technique aimed to create a more homogeneous texture on the lithium surface, which is beneficial for mitigating dendrite formation during battery cycling. The third protection strategy involved coating the lithium sheet with a thin, uniform layer of PLA[7]. The PLA was applied evenly and subsequently subjected to a heating process to ensure proper adhesion and uniform coating of the lithium surface (Fig. S3c). This protective coating serves as a barrier to reduce unwanted chemical reactions with the electrolyte, thereby improving the long-term stability of the lithium anode. Fig. S3d-f present SEM images of the treated lithium electrode prior to initiating the battery cycling tests. These images illustrate the morphological changes induced by each protection strategy, highlighting the improved surface characteristics that are expected to mitigate the issues commonly associated with lithium metal anodes, such as dendrite growth and surface inhomogeneities.

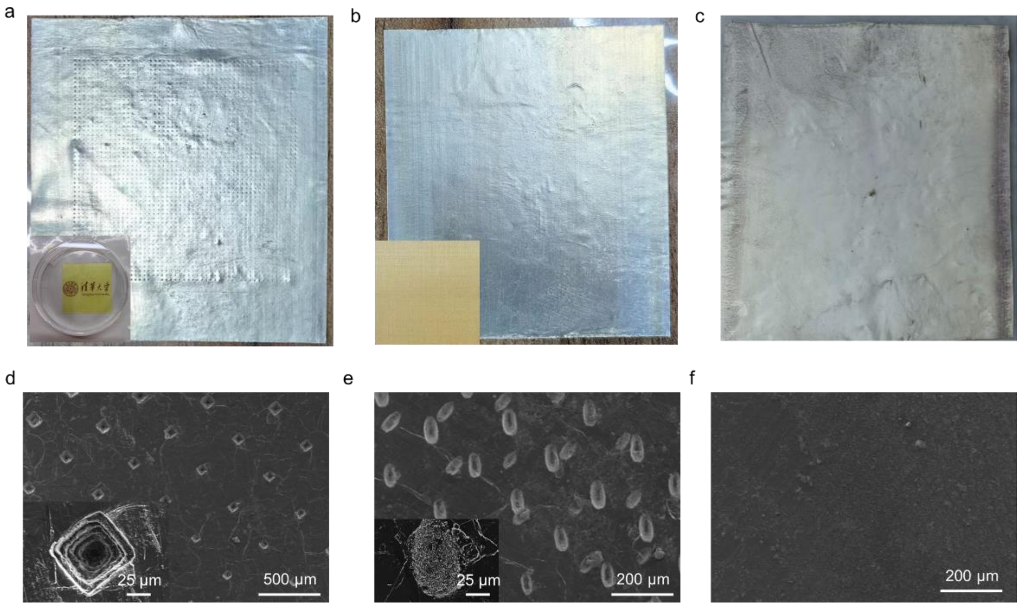

Fig. S3 | a-c photos and **d-f** SEM images of Pyramid-patterned Li, Cu-patterned Li, and PLA-Li.

Anode morphology of Bare-Li

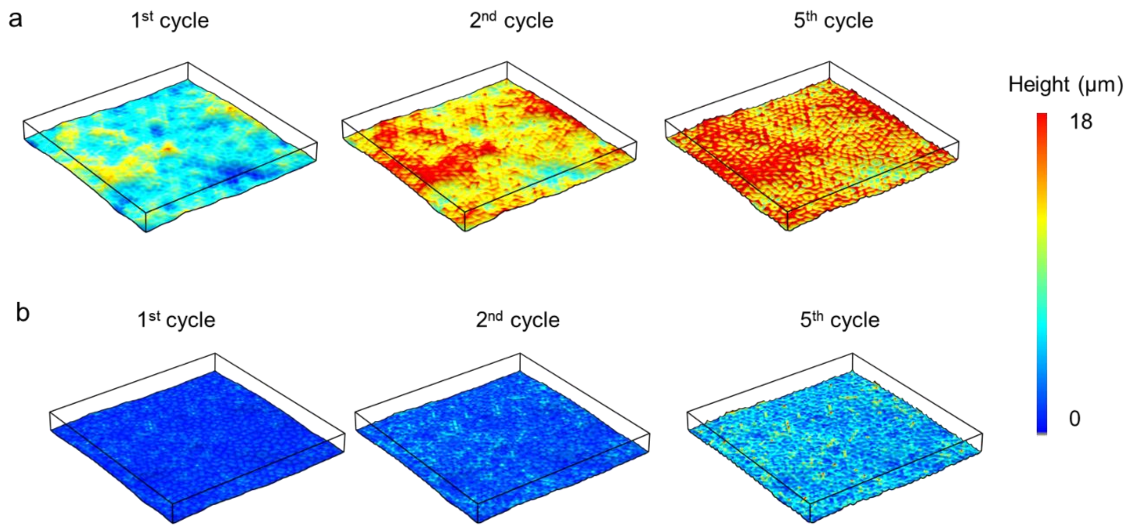

Fig. S4 | Simulations of Bare-Li anode morphology. **a** charge process, and **b** discharge process.

Electrochemical impedance spectroscopy (EIS) of the button cells

The resistance values of these electrodes were measured using EIS, and the corresponding results are shown in Fig. S5. According to post-cycling results, Bare-Li exhibits high interfacial and charge transfer resistances, which produce significant Joule heating during subsequent charge-discharge processes. This shortens the battery's cycle life by accelerating the breakdown of internal chemical components and causing heat accumulation. On the other hand, after the same number of cycles, anodes shielded by different tactics show noticeably lower resistance values. The PLA-Li anode significantly extends the lifecycle of LMBs by demonstrating exceptional interfacial stability and charge transfer efficiency, which effectively reduces cell deterioration and heat accumulation.

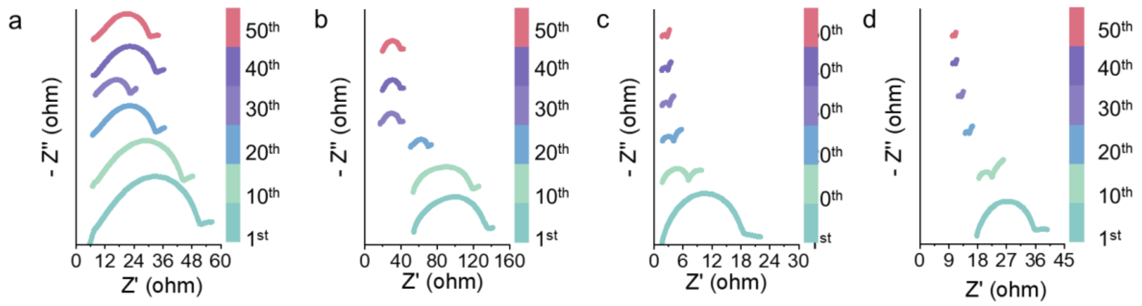

Fig. S5 | Electrochemical impedance spectroscopy (EIS) of **a** Bare-Li, **b** Pyramid-patterned Li, **c** Cu-patterned Li, and **d** PLA-Li.

Response spectra of SMF to temperature

Through post-processing of the data, the temperature response at various positions along the optical fiber is illustrated in Fig. S6. This figure shows the temperature variations at different locations along the length of the fiber, reflecting the dynamic response of the optical fiber to thermal conditions, with a spatial resolution of 1 mm. The pouch cell's lifecycle consists of 100 full charge-discharge cycles, with temperature data recorded every ten cycles. It can be observed that during the entire lifecycle, the temperature shows an overall upward trend. The untreated Li anode exhibits numerous hotspots (Fig. S6a), whereas after treatment with pyramid structures, Cu-patterned, and PLA, the number of hotspots decreases, with some instances showing temperatures returning to their initial levels (Fig. S6b-d). This demonstrates that the uniformity of Li ion deposition significantly influences hotspot accumulation, and the treatment methods improve the uniformity of deposition, thereby reducing hotspot formation.

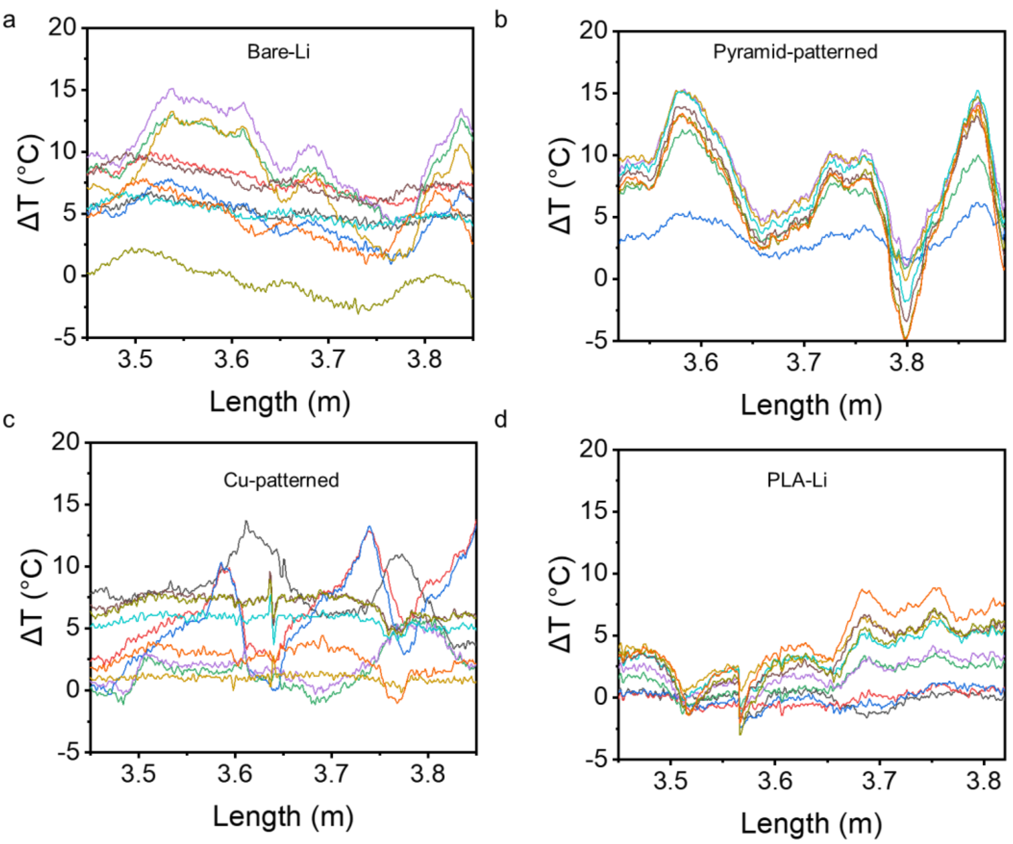

Fig. S6 | Response spectra of SMF to temperature of **a** Bare-Li, **b** Pyramid-patterned Li, **c** Cu-patterned Li, and **d** PLA-Li.

Temperature distribution of Li electrodes

The following figures (Fig. S7-S10) show the temperature distribution map of unprotected and protected lithium metal electrodes under three different protection strategies during cycling. Specifically, it illustrates the 5th cycle, the cycle before capacity fading, the moment of capacity fading, and the 100th cycle. The temperature distribution is presented through processed Archimedean spiral curves and super-resolution images.

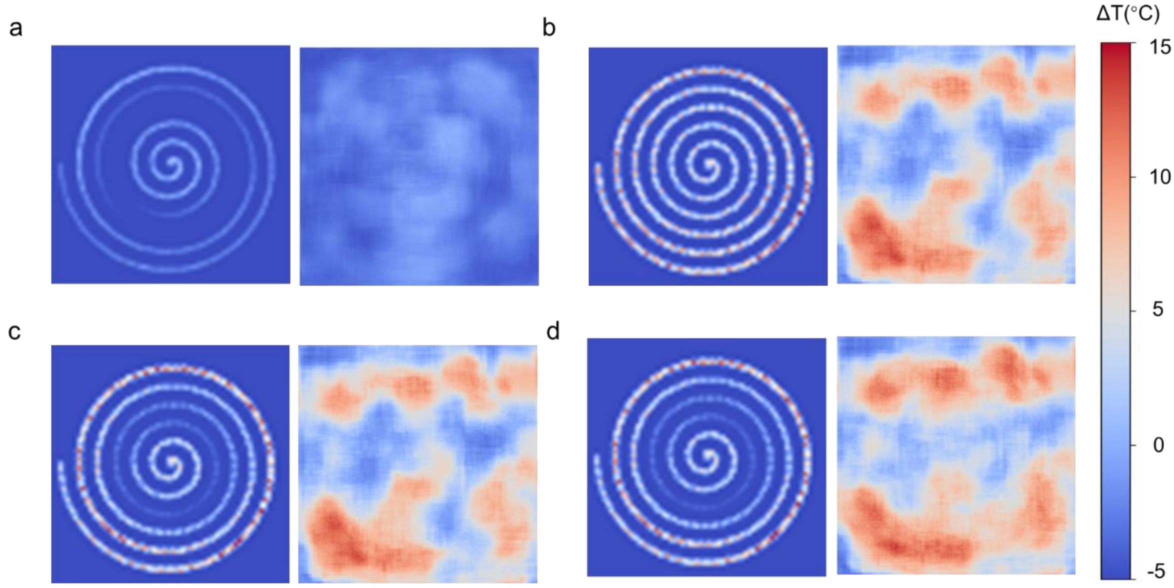

Fig. S7 | Low-resolution and super-resolution temperature maps of bare-Li at: a 5th cycle, **b** 47th cycle **c** 48th cycle, and **d** 100th cycle.

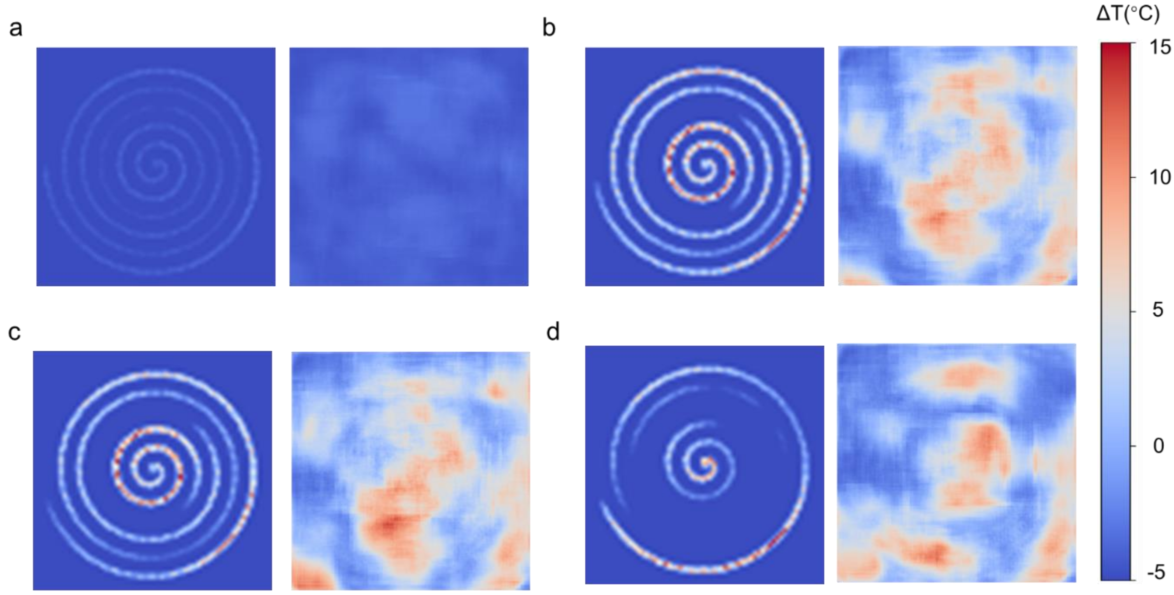

Fig. S8 | Low-resolution and super-resolution temperature maps of Pyramid-patterned Li at: a 5th cycle, **b** 55th cycle **c** 56th cycle, and **d** 100th cycle.

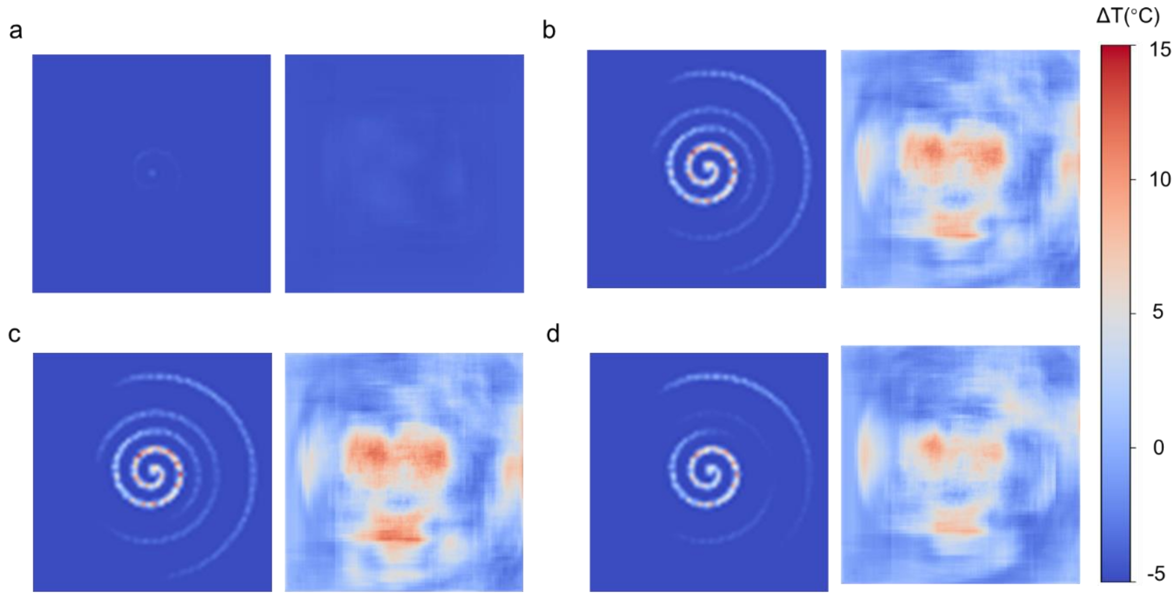

Fig. S9 | Low-resolution and super-resolution temperature maps of Cu-patterned Li at: a 5th cycle, **b** 59th cycle **c** 60th cycle, and **d** 100th cycle.

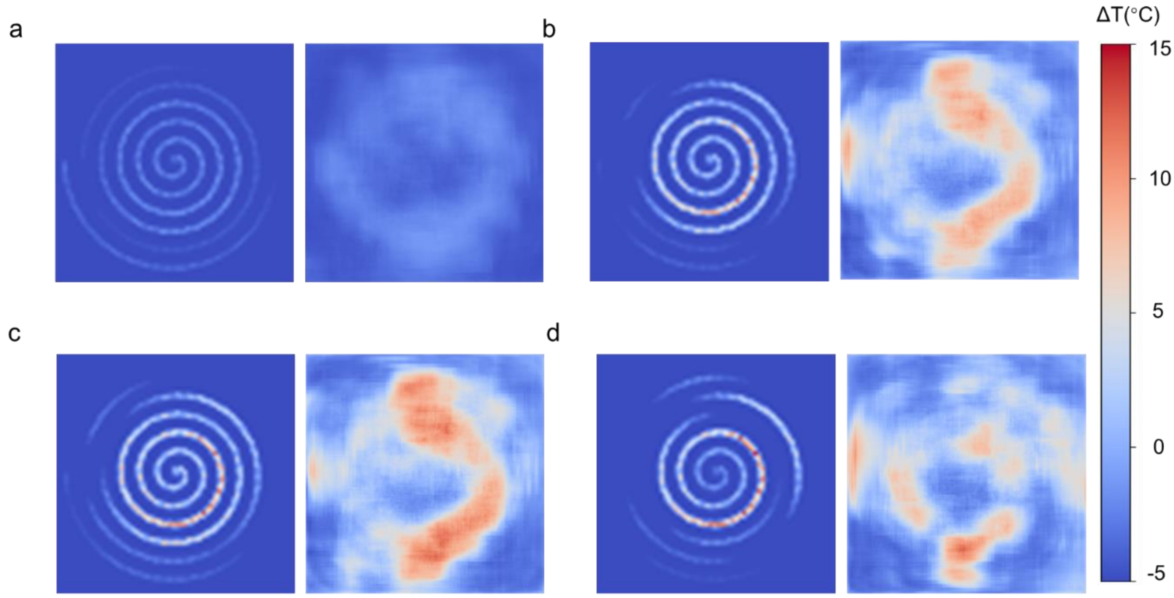

Fig. S10 | Low-resolution and super-resolution temperature maps of PLA- Li at: a 5th cycle, **b** 69th cycle **c** 70^{h} cycle, and **d** 100th cycle.

Table. S1 The electrodes parameter of button cells and the formation conditions.

**Parameter**	**Anode**	**Cathode**
Material	Bare-Li	$LiFePO_4$
	Pyramid-patterned Li	
	Cu-patterned Li	
	PLA-Li	
Separator	Celgard 2500	
Electrode Size	Diameter: 18 mm	Diameter: 12 mm
Mass Loading		10.52 mg/cm^2

Table. S2 The electrodes parameter of pouch cells and the formation conditions.

Parameter	Anode	Cathode
Material	Bare-Li	$LiFePO_4$
	Pyramid-patterned Li	
	Cu-patterned Li	
	PLA-Li	
Separator	Celgard 2500	
Electrode Size	65 mm*60 mm	65 mm*60 mm
Mass Loading		400 mg

SRGAN model

Specifically, we evaluated input images of sizes 32×32, 64×64, and 128×128, while adjusting only the final upsampling factor in the model to ensure proper functionality (Table. S3) of Mean Absolute Error (MAE), and Mean Squared Error (MSE). Our findings suggest that larger input sizes lead to degraded performance. This can be attributed to the fact that the model was trained with smaller input sizes, making it susceptible to information overload and noise when processing larger inputs. Addressing this issue would require a deeper network to enhance performance. However, in practical applications, both the battery size and fiber distribution are fixed, meaning that the input size remains constant. Therefore, training an optimized model based on this fixed input size is sufficient for our use case.

Table. S3 Quantitative error analysis for both the center and boundary regions with different input image sizes.

	PSNR (dB)	SSIM	MAE	MSE
Center (64*64)	22.55	0.6718	0.0771	0.0171
Boundary (64*64)	21.03	0.6684	0.0779	0.0134
Whole (64*64)	21.36	0.6232	0.0776	0.0148
Center (32*32)	25.27	0.7461	0.0544	0.0078
Boundary (32*32)	24.82	0.7457	0.0532	0.0063
Whole (32*32)	24.88	0.7069	0.0536	0.0069
Center (128*128)	18.45	0.5716	0.1076	0.0239
Boundary (128*128)	16.06	0.5573	0.1271	0.0293
Whole (128*128)	16.60	0.4998	0.1199	0.0273

Reference

1. Qu S, Xu Y and Huang S *et al.* Recent Advancements in optical frequency-domain reflectometry: a review. *IEEE Sens J* 2023; **23**: 1707-1723.
2. Ding Z, Guo H anda Liu K *et al.* Advances in distributed optical fiber sensors based on optical frequency-domain reflectometry: a review. *IEEE Sens J* 2023; **23**: 26925-26941.
3. Yuksel K, Wuilpart M and Moeyaert V *et al*. Original monitoring technique for passive optical networks combining fiber Bragg gratings and wavelength swept light source. *IEEE* 2010: 1-4.
4. Park J, Jeong J and Lee Y *et al.* Micro□patterned lithium metal anodes with suppressed dendrite formation for post lithium□ion batteries. *Adv Mater Interfaces* 2016; **3**: 1600140.
5. Wang H, Li J and Huang Y *et al*. Engineering array-patterned cathodes and anodes for synergistically enabling high-performance lithium metal batteries. *ACS Appl Mater Interfaces* 2023; **15**: 15525-15532.
6. Wang H, Hu P and Liu X *et al.* Sowing Silver Seeds within Patterned Ditches for Dendrite□Free Lithium Metal Batteries. *Adv Sci* 2021; **8**: 2100684.
7. Chang C, Zhang M and Lao Z *et al.* Achieving Stable Lithium Anodes through Leveraging Inevitable Stress Variations via Adaptive Piezoelectric Effect. *Adv Mater* 2024; **36**: 2313525.
