# Supplementary material for: Operando spatiotemporal super-resolution of thermal events monitoring in lithium metal batteries
Source: Natl Sci Rev. 2025 Mar 6;12(5):nwaf088. doi: 10.1093/nsr/nwaf088 (PMC11987594; doi:10.1093/nsr/nwaf088)
Supplement: nwaf088_Supplemental_File [file nwaf088_supplemental_file.zip › Teaser text.docx]

This study presents the first operando spatiotemporal super-resolution thermal monitoring system for lithium metal batteries, offering unprecedented spatial and temporal precision to enhance safety, verify capacity degradation and optimize thermal management strategies.
